# Treatment concerns for psychiatric symptoms in patients with COVID-19 with or without psychiatric disorders

**DOI:** 10.1192/bjp.2020.84

**Published:** 2020-04-09

**Authors:** Kai Zhang, Xiaoqin Zhou, Huanzhong Liu, Kenji Hashimoto

**Affiliations:** 1 Department of Psychiatry, Chaohu Hospital, Anhui Medical University, China; 2 Division of Clinical Neuroscience, Chiba University Center for Forensic Mental Health, Japan

**Keywords:** Antipsychotics, antiviral, psychotic disorders, coronavirus, drug interactions and side-effects

## Abstract

Many psychiatric patients have been infected with COVID-19, and patients with COVID-19 may develop psychiatric symptoms after treatment with antiviral drugs. Given the tolerability and minimal P450 interactions, antidepressants (i.e., citalopram, escitalopram etc.), antipsychotics (i.e., olanzapine) and valproate can be considered to be safe in combination with antiviral drugs.

Since December 2019, the novel coronavirus disease 2019 (COVID-19) has spread from Wuhan to other cities in China and around the world, including Japan.^1^ As of 17 February 2020, there have been 70 673 confirmed cases in China. The National Health Commission of China (NHCC) has published guidelines for treatment of COVID-19. Recommended antiviral drugs by the NHCC include interferon-α, lopinavir/ritonavir and ribavirin.^2^

As psychiatrists, we are particularly concerned about two aspects of this situation. First, many patients with psychiatric disorders have been infected with COVID-19, and second, patients with COVID-19 infections may develop a number of psychiatric symptoms, including anxiety, fear, depression and insomnia, after treatment with antiviral drugs. This is concerning because Wuhan Mental Health Center, the largest psychiatric hospital in Hubei province, reports that more than 80 staff and patients have been infected with COVID-19.^3^

If a patient with psychiatric disorder is infected with COVID-19, antiviral drugs must be used in combination with psychotropic drugs, including antipsychotic, antidepressant and antianxiety drugs. If antiviral drugs are used without supplemental medication, patients with psychiatric disorders can experience relapses in their mental illness. Importantly, patients may present with impulsivity, running or other abnormal behaviours that are not conducive to the control of COVID-19. Psychotropic medications are thus necessary to avoid these and other behavioural problems. The most pressing question for doctors on the front line is how to choose the appropriate psychotropic drug in combination with the antivirals recommended by the NHCC.

The combination of antivirals and psychotropic drugs should be considered in the context of potential drug–drug interactions. Most antipsychotic drugs and antiviral medications utilise cytochrome P450 (CYP) enzymes for their metabolism.^4^ Anxiety, nervousness, insomnia and other symptoms can occur in patients with COVID-19 because of stressor events. Because sedative and hypnotic drugs such as oxazepam and lorazepam are not metabolised by the CYP system, these are quite safe when used in combination with the recommended antiviral drugs.

Given the tolerability and minimal P450 interactions, antidepressants (citalopram, escitalopram, etc.), antipsychotics (olanzapine) and valproate can be considered to be safe in combination with antiviral drugs. Special care should be taken to ensure that drug–drug interactions are prevented when psychotropic drugs are used in combination with antivirals.

## Supplementary material

For supplementary material accompanying this paper visit https://doi.org/10.1192/bjp.2020.84. click here to view supplementary material

## References

[1] Wang C, Horby PW, Hayden FG, Gao GF. A novel coronavirus outbreak of global health concern. Lancet 2020; 395(10223): 470–3. 31986257 10.1016/S0140-6736(20)30185-9 PMC7135038

[2] National Health Commission of the People's Republic of China (NHCC). Diagnosis and Treatment Guideline of COVID-19 (Version 5). NHCC, 2020 (http://www.gov.cn/zhengce/zhengceku/2020-02/09/content_5476407.htm). 10.21147/j.issn.1000-9604.2020.04.01 PMC7491549 32965276

[3] Wu XY. About 80 doctors and patients at the Wuhan Mental Health Center were diagnosed with COVID-19. China News Weekly, 2020 (http://society.people.com.cn/n1/2020/0209/c1008-31577664.html).

[4] Roncero C, Villegas JL, Martinez-Rebollar M, Buti M. The pharmacological interactions between direct-acting antivirals for the treatment of chronic hepatitis C and psychotropic drugs. Expert Rev Clin Pharmacol 2018; 11(10): 999–1030. 30199279 10.1080/17512433.2018.1519392

